# Hip Arthroplasty in a Patient with Transfemoral Amputation: A New Tip

**DOI:** 10.1155/2015/593747

**Published:** 2015-02-08

**Authors:** Hassan Boussakri, Ihab Alassaf, Samir Hamoudi, Abdelhalim Elibrahimi, Philbert Ntarataz, Abdelmajid ELMrini, Jean Francois Dumez

**Affiliations:** ^1^Orthopedics and Traumatology Surgery Department (AP4), Moulins Hospital, 03000 Moulins, France; ^2^Department of Orthopaedic Surgery (B4), CHU Hassan II Hospital, University of Sidi Mohamed Ben Abdellah, 3000 Fez, Morocco

## Abstract

Femoral fractures in amputation stump are challenging injuries to manage. The authors describe a case of a 51-year-old patient with a right above knee amputation, who had a right hip femoral neck fracture. In this technical note, we describe a technical and surgical procedure with intraoperative tips and tricks, in which we use commonly available materials, for the safe management in such clinical situations.

## 1. Introduction

Residual limb fracture in an amputee is not a common entity and there are few publications regarding this pathology [1]. Therefore, there are few pieces of information in the specialty literature regarding the management of femoral neck fractures in patients with ipsilateral above or below knee amputations. Hip fractures are a common source of morbidity and mortality worldwide [2], but hip arthroplasty has totally changed the prognosis of femoral neck fractures. Femoral neck fractures in transfemoral amputation poses a problem of choice of the surgical approach and technique, because stump manipulation and positioning of the affected side of the patients with above or below the knee amputations represent a significant challenge. In our case, treatment strategy is made by a team of experienced surgeons after a preoperative planning.

We present an unusual case of bipolar hemiarthroplasty for a subcapital femoral fracture in a patient with an above knee amputation of the same extremity. In this technical note, we describe the positioning technique, the surgical approach, and a technical variation that streamlines the surgical procedure and uses commonly available materials. This simple trick makes the procedure and the manipulation of the stump easier and avoids invasive procedures.

## 2. Case Report

A 81-year-old female had a transfemoral (above knee) amputation of the right limb at the age of 70, as a result of the failure of the femoropopliteal artery bypass realized twenty years before in our hospital. She received a prosthesis which provided sufficient walking autonomy. Besides her disability resulting from the amputation, she also suffered from thoracic and lumbar spondylosis with vertebral insufficiency. She had essential arterial hypertension, chronic atrial fibrillation, osteoporosis under treatment, and bilateral surgical treatment of cataract.

**Figure 1:**
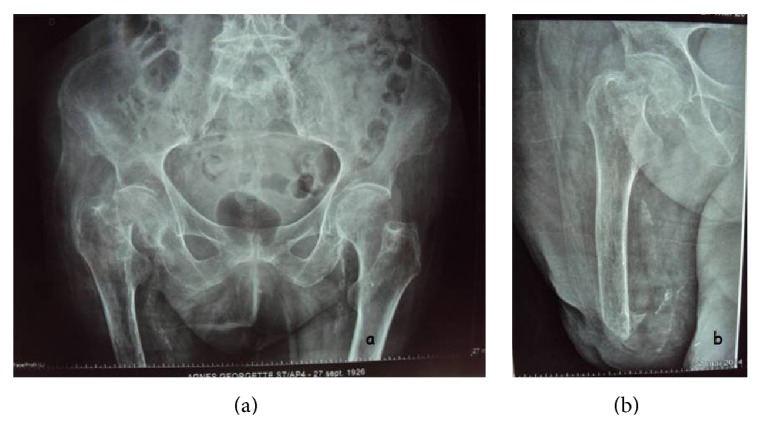
The X-ray of the pelvis and the hip (an anteroposterior X-ray) showing a fracture of the femoral neck.

The patient was presented to the emergency department with a painful right hip following falling on that side. Clinical examination found pain in the right hip, localized pain at palpation of the greater trochanter, and impossible standing position. Radiological examination found a subcapital femoral neck fracture of the right hip (Figures 1(a) and 1(b)). The patient was treated in our Orthopedics and Traumatology Surgery Department by hip hemiarthroplasty with physiotherapy.

### 2.1. Surgical Procedure

**Figure 2:**
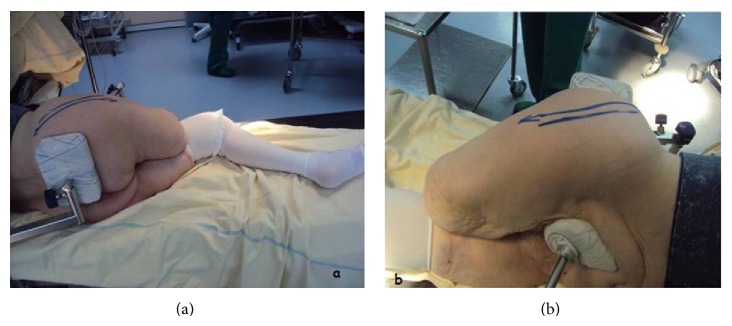
Intraoperative photographs demonstrating the setup of the patient.

**Figure 3:**
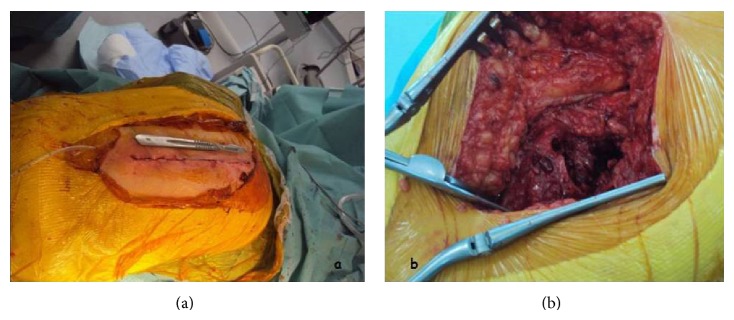
A clinical picture has showed the Hardinge approach. (a) The skin incision. (b) Articular exposure.

Under epidural anesthesia, the patient was placed in the lateral decubitus (Figures 2(a) and 2(b)), and a classical Hardinge surgical approach was used [3]. Skin incision was about 90 mm from proximal to distal, performed along the midline of the greater trochanter (Figure 3(a)). The subcutaneous fat and proximal fascia lata were incised, exposing the gluteus maximus muscle. Separation of the gluteus maximus fibers exposed the gluteus medius and minimus muscles. The gluteus minimus was sectioned exposing the anterior hip capsule and the ligament system underneath. Following the anterior dissection along the greater trochanter and on the femoral neck which leads to the capsule, gluteus minimus needed to be released from the anterior greater trochanter. The vastus lateralis tendon was not incised, and the anterior hip capsule was exposed. Distally the incision passed down to the bone through the vastus lateralis near the anterior surface of the femur (Figure 3(b)).

**Figure 4:**
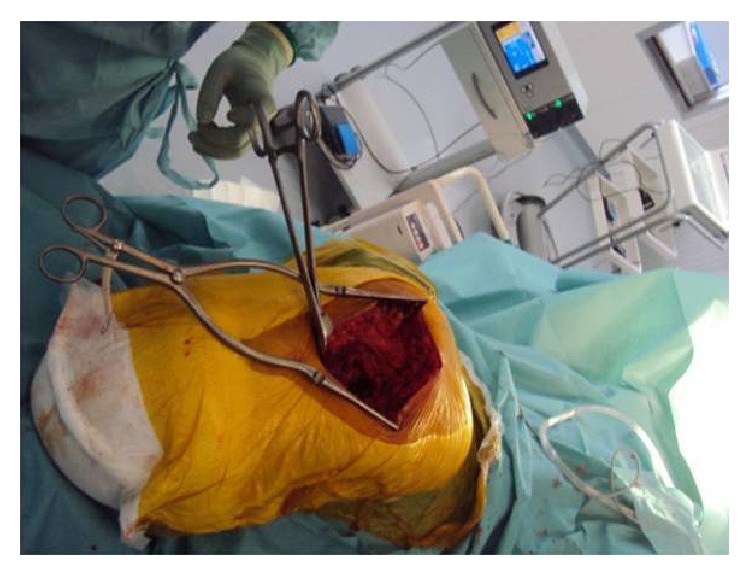
A bone forceps placed 5 cm under the trochanter.

It is difficult to realize the dislocation of the femoral head and manipulate the amputation stump because of the short lever arm of the affected femur. We describe a simple but reproducible technique. The required materials are available in all orthopedics operating rooms. We placed a bone forceps, 5 cm, under the lesser trochanter to the femoral shaft benefiting of the already existing incision of the used surgical approach. This simple trick makes the procedure easier and avoids an invasive procedure, by manipulating internally the femur during the procedure due to posterior dislocation of the joint (Figure 4).

From the trochanteric fossae, a 45° angle was marked regarding the femoral shaft axis and femoral neck osteotomy was performed. The femoral head was removed by a “corkscrew” with retracting the distal femur using a bone forceps.

**Figure 5:**
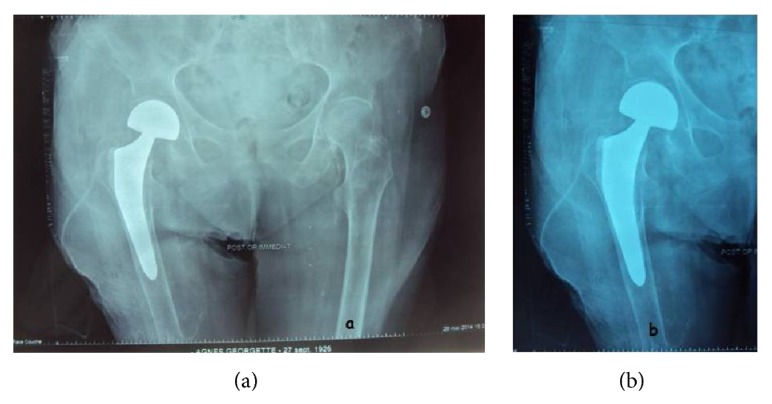
Postoperative radiography with uncemented bipolar hemiarthroplasty.

For maintaining the desired anteversion while preparing the femoral medullar canal with rasps, we used a bone forceps. The femoral canal was prepared according to standard technique. Bipolar hemiarthroplasty was inserted in correct anteversion. It must be placed in the appropriate, predetermined depth. We used trial femoral head prosthesis on the stem to confirm both diameter and length of neck. We attached the definitive femoral head to the stem and reduced the hip (Figure 5). The wound was closed as usual, but taking special care to close the fascia properly (Figure 3(a)).

**Figure 6:**
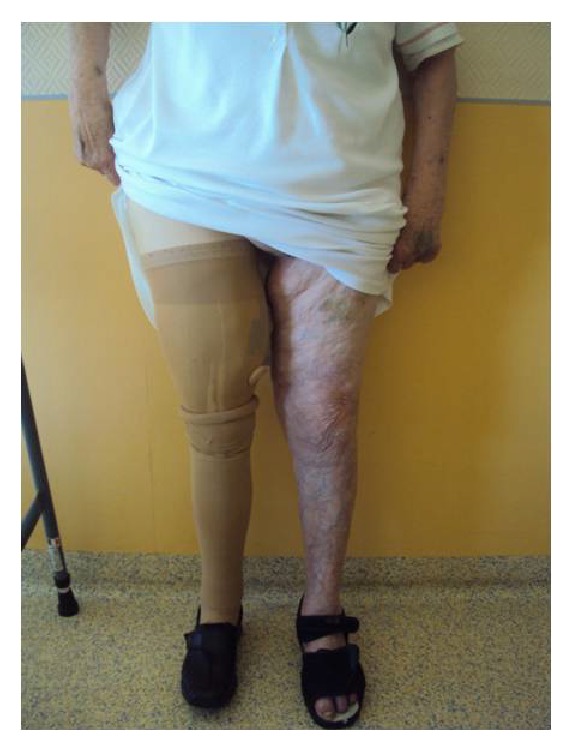
Satisfactory functional results, at the follow-up of 24 months.

**Figure 7:**
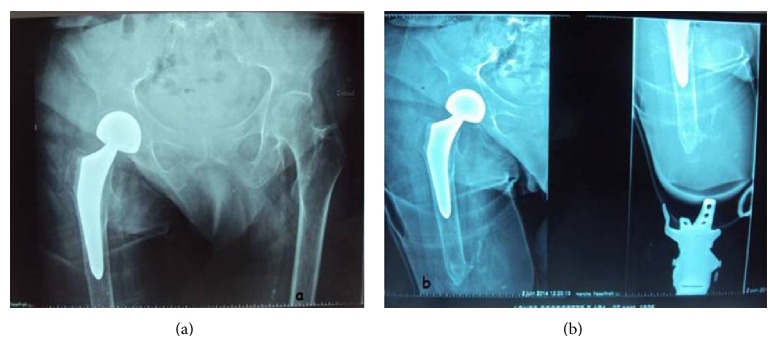
Pelvis and hip radiographies (an anteroposterior X-ray), at the follow-up of 24 months after the hemiarthroplasty.

30 months after the surgery, the patient was walking well using prosthesis. On examination she had a full range of motion, without any pain, and no leg length difference was noted (Figure 6). The X-rays of the pelvis and the hip showed a normal bipolar hemiarthroplasty (Figures 7(a) and 7(b)).

## 3. Discussion

Arthroplasty of the hip represents an operation that is used most commonly to treat a fracture of the hip. These fractures are a common source of morbidity and mortality worldwide [2], but hip arthroplasty has totally changed the prognosis of the femoral neck fracture. Residual limb fracture in an amputee is not a common entity and there are few publications regarding this pathology [1, 4]. Pyka and Lipscomb [5] found that the prosthesis was the direct cause in 7 out of 11 lower extremity fractures among 14 amputees that have been reported. We believe that this finding is explained by osteoporosis, but especially by the lever arm carried by the prosthesis. The fractures of the femoral neck in transfemoral amputation affect old osteoporotic patients. This affects the therapeutic management and causes incidents such as fractures of amputation stump.

There are a few documents which were previously reported, but neither of them contained information regarding the management of hip neck fractures in patients with ipsilateral above or below knee amputations, to the best of our knowledge. Some authors suggest that most of the amputees with fracture could be treated conservatively, as the use of these methods avoids malunion and intraarticular complications [6].

Concerning the surgical treatment, the choice of patient positioning and manipulating of the lower limb poses a significant challenge. The surgical procedure must take into consideration certain factors such as age of patient, operative time of the surgery, surgical approach allowing better exposure, and risk of fracture related to osteoporosis. Limbs that have undergone an above knee amputation can have short stumps that can be very challenging to position or apply any manipulation. To respond to this different specification we chose the surgical approach of Hardinge [3], which has as an advantage: better exposure for orientation of the implant. The described surgical technique has the advantage of being simple and easy to carry out and requires only a material, which is available in any orthopedics operating room. The combination with Surgical Hardinge approach allows it to be less traumatic and provides rotation and traction of the stumps, thereby also reducing the surgery time, compared to other surgical approaches: Moore and Kocher-Langenbeck posterolateral approach. Therefore, to avoid using other complex techniques such as the use of the skeletal pin is a clever trick. Application of such a pin carries with it the risk of injury to the soft tissues of the stump. The pin may pull out the osteoporotic bone on application of traction with fracture risk stump. Concerning the postoperative period, Bowker et al. [7] felt that properly closed operative wounds did not lead to troublesome scar in amputees, and easily workable plastic in modern prosthesis and fixation devices presented a minimum problem after internal fixation.

Although the problem of aging cannot be changed, a realistic attitude could prevent accidental fall in elderly amputees. The high risk of sustaining a fracture in osteoporotic residual stump skeleton should be emphasized to these patients as part of health education.

## 4. Conclusion

We report a rare case of fracture of the femoral neck on a transfemoral amputation treated by hemiarthroplasty. There is little information in the specialized literature regarding this entity. The surgical technique described here has the advantage of being simple, easy to carry out and requires only a bone forceps, which is available in any operating room. To avoid having an invasive procedure is a clever trick, and it makes a complex and difficult operation much easier.
